# The Critical Role of Inhibitor of Differentiation 4 in Breast Cancer: From Mammary Gland Development to Tumor Progression

**DOI:** 10.1002/cam4.70856

**Published:** 2025-04-05

**Authors:** Yuhang Song, Panshi Zhang, Sudhanshu Bhushan, Xinhong Wu, Hongmei Zheng, Yalong Yang

**Affiliations:** ^1^ Department of Breast Surgery Hubei Cancer Hospital, Tongji Medical College, Huazhong University of Science and Technology, Hubei Provincial Clinical Research Center for Breast Cancer, Wuhan Clinical Research Center for Breast Cancer; National key clinical specialty construction discipline Wuhan Hubei China; ^2^ Department of Thyroid and Breast Surgery Tongji Hospital of Huazhong University of Science and Technology Wuhan Hubei China; ^3^ Department of Anatomy and Cell Biology Unit of Reproductive Biology, Justus‐Liebig‐University Giessen Giessen Germany

**Keywords:** breast cancer, development, ID4, mammary gland

## Abstract

Inhibitor of differentiation 4 (ID4) is a highly conserved DNA‐binding inhibitory protein of mammals, and its main role is to bind basic helix–loop–helix (b‐HLH) so that it loses its DNA‐binding activity, which in turn regulates the transcription of key genes, regulating cell differentiation and proliferation as the physiological function. Breast tissue is a highly heterogeneous tissue organ with a strong capacity for remodeling and differentiation, and studies of breast carcinogenesis suggest that the mechanisms regulating the differentiation of breast tissue interact critically with tumorigenesis. The expression level of ID4 and its regulatory mechanism play a crucial role in the study of breast cancer, but its oncogenic or oncostatic role has not yet been unanimously identified, and its regulatory mechanism in breast cancer still needs to be further elucidated. This review summarizes and analyzes the relevant studies of ID4 and the research progress in breast cancer, integrating the development of breast tissue and tumorigenesis with the regulatory role of ID4, to provide some insights into develop new treatment strategies and diagnostic biomarkers.

**Abbreviations**
bc
: breast cancer
BLBC: basal‐like breast cancer
CBF1: C‐promoter binding factor‐1
CSC: cancer stem cell
DFS: disease‐free survival
ER: estrogen receptor
FOXA1: Forkhead Box Protein A1
ID4: inhibitor of differentiation 4
KO: knock out
MDC1: mediator of DNA damage checkpoint protein 1
OS: overall survival
PR: progesterone receptor
TEBs: terminal end buds
TNBC: triple‐negative breast cancer
WT: wild‐type

## 1 Introduction

ID4 (inhibitor of differentiation 4, also known as an inhibitor of DNA‐binding 4) is a transcriptional regulator that plays a critical role in various cellular processes, including cell differentiation, proliferation, and apoptosis [1]. ID4 was found to be mostly expressed in cells with stem properties or low degrees of differentiation, especially in embryonic tissues, which decreases with the differentiation of organ tissues [2, 3, 4]. In many disease models, including tumors, researchers have found that the expression level of ID4 is closely associated with tumor incidence, prognosis, and other important indicators, especially in prostate cancer, where *ID4* is considered to be an independent and crucial oncogene [4, 5, 6, 7, 8].

Breast cancer (BC) has surpassed lung cancer to become the most prevalent malignant tumor affecting human health [9]. With advances in molecular phenotyping, a better understanding of the regulatory mechanisms of the immune microenvironment, and comprehensive analyses of breast cancer genomics, researchers have gradually uncovered key aspects of breast cancer development [10, 11, 12, 13]. However, the key mechanisms of BC are still to be elucidated, especially in triple‐negative breast cancer (TNBC), and accumulating data indicate that the development of BC is, to a certain extent, related to the dysregulation of stem cells in the mammary gland [14, 15, 16, 17, 18, 19].

This review provides insights into the research progress of ID4 in mammary gland development and breast carcinogenesis from the perspective of development and further discusses the potential of ID4 as a target for intervention of key factors in BC pathogenesis.

## 2 Basic Physiological Functions of ID4

**FIGURE 1:**
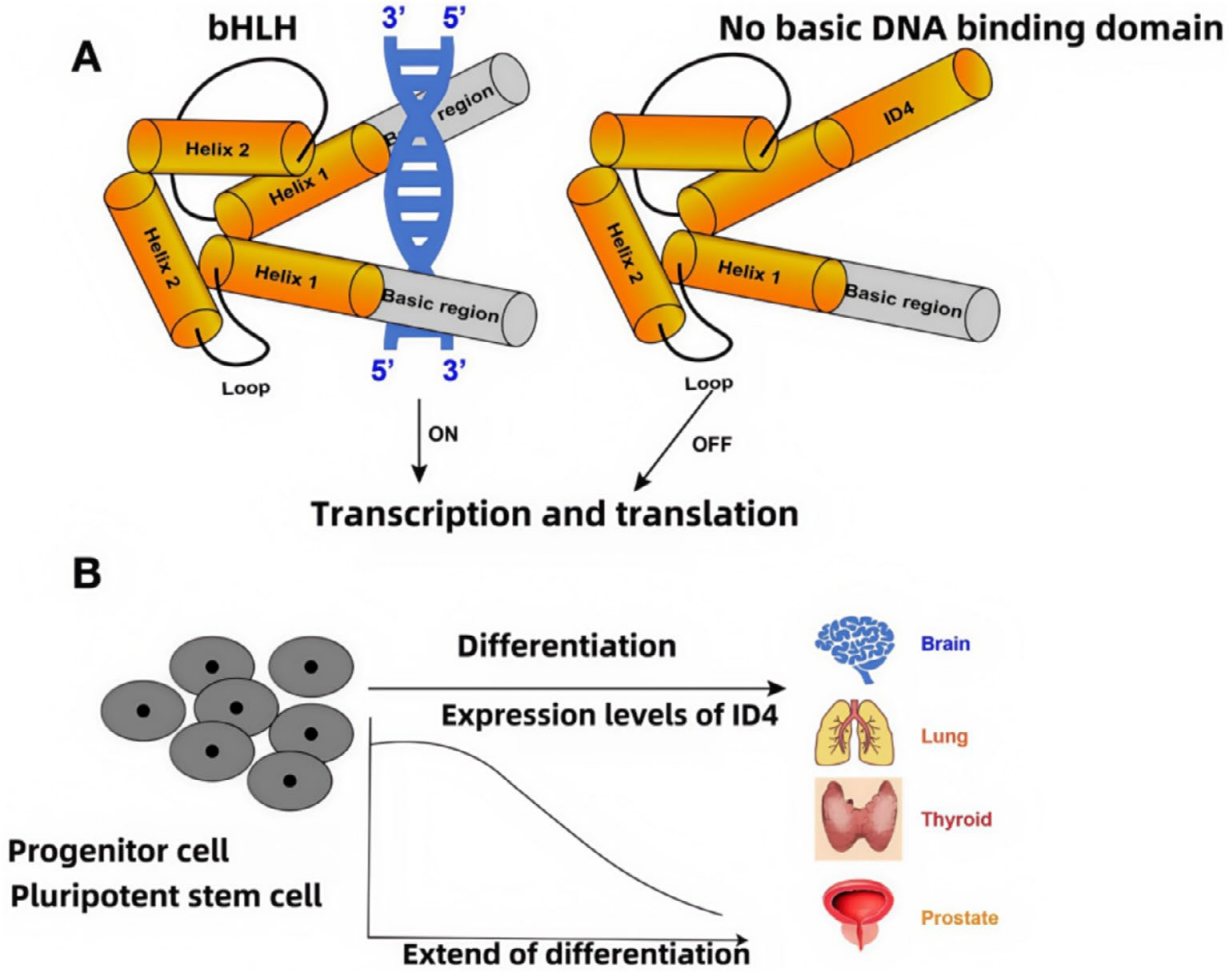
(A) The structure and basic physiological functions of b‐HLH and ID4. The left part of the figure shows the structure of b‐HLH, which consists of the basic region and the HLH region. The basic region serves as a DNA‐binding region that recognizes the E‐box and the G‐box. The right part of the figure shows that ID4 is characterized by a typical HLH domain comprising two α‐helices connected by a loop. ID4 does not contain the basic DNA binding domain and inactivates the transcriptional activity of b‐HLH proteins by forming inactive heterodimeric complexes with their b‐HLH partners. (B) The expression level of ID4 gradually decreases as the differentiation of embryonic cells increases. The expression level of ID4 gradually decreases as the differentiation of embryonic cells increases. Created in https://BioRender.com.

IDs induce cell proliferation, and ID1 antagonizes the action of E proteins, whereas ID2 and ID3 act by binding to pRB (in retinoblastoma) and related proteins, p107 and p130 [20, 21, 22]. These three ID proteins have been described in other reviews [23, 24, 25] and will not be listed here. ID4 is a transcriptional regulator that belongs to the helix–loop–helix (HLH) family, characterized by a typical HLH domain comprising two α‐helices connected by a loop. The two helices generally twist in the same direction and form a specific spatial configuration. The basic‐HLH (b‐HLH) transcription factors contain two highly conserved and functionally distinct structural domains: the basic region and the HLH region [2]. The basic region consists of 10–20 amino acids and is located at the N‐terminal end, which serves as a DNA‐binding region that recognizes the E‐box and the G‐box. The HLH region located at the C‐terminal end relies on the interactions of hydrophobic amino acids to form a homo‐ or heterodimer of two HLH proteins, which then regulate the expression of downstream target genes [2, 26]. ID4 does not contain the basic DNA‐binding domain and inactivates the transcriptional activity of b‐HLH proteins by forming inactive heterodimeric complexes with their b‐HLH partners [4, 27]. This property has led them to be called DNA‐binding inhibitors (shown in Figure 1A).

This review focuses primarily on ID4, not only because ID4 is regulated in a very different manner from the other three IDs, but also because the expression of ID4 shows a close correlation with diseases and also displays a unique negative regulation on ID1/2/3 [28, 29]. Unlike ID1/2/3, which have overlapping expression in embryonic tissues, ID4 is predominantly expressed in the mesodermal part of the embryo [28]. At a particular time point in embryonic development, ID proteins are expressed in all cell lines, and the expression level of ID4 gradually decreases as the differentiation of embryonic cells increases [23, 24, 28]. In human fetal tissues, ID4 is highly expressed in the brain, lung, and kidney, but not in the liver [4, 28]. In adult tissues, it is expressed predominantly in the thyroid gland and, to a lesser extent, but also highly expressed in the brain and testis [28]. Studies indicated that ID4 is highly expressed in osteoblasts, prostate epithelial cells, neurons in the central nervous system, sertoli cells in the testis, and glial cells in the brain, supporting its role as a pro‐differentiation factor [20, 28, 30] (shown in Figure 1B).

## 3 The Roles of ID4 in Normal Tissues

By developing a mouse global *Id4*‐KO (knock out) model, the researchers found at least 50% embryonic lethality, significant weight loss in born purebred mice, and less than 20% survived to adulthood, and all surviving mice developed adipose dysplasia and osteoporosis [31, 32]. Depletion of *Id4* also induced slower proliferation of neural precursor cells than wild‐type (WT) counterparts, and spermatogenesis was significantly impaired in adulthood [30, 32]. Mice with *Id4*‐KO exhibited obvious osteoporosis, as evidenced by a significant reduction in osteoblast differentiation, the main mechanism being that normal Id4 promotes osteoblast differentiation by releasing Hes1 through the promotion of the Hes1‐Hey2 complex [33] (Table 1). In addition to adjunctive validation of the physiological role of Id4 in promoting tissue differentiation through gene knockdown, researchers have also found, through functional experiments, that Id4 inhibits oligodendrocyte differentiation by inhibiting the transcriptional activity of *Olig1/2* through binding to b‐HLH [34]. An in vivo test also showed that one of the mechanisms by which Id4 may regulate normal prostate development is through regulating androgen receptor binding to respective response elements such as those on NKX3.1 promoter [35]. *Nkx3.1* regulates early postnatal ductal morphogenesis and maintains normal differentiation of the prostate epithelium, including the production of secretory proteins [39, 40]. Taken together with the accumulating evidence described above, we can see that Id4 has important regulatory roles in tissue and cellular differentiation.

**TABLE 1:** The different roles of ID4 in normal tissues and cancer cells.

Normal tissues or cancer cells	Role	Mechanism	References
Bone	Promotes osteoblast differentiation	Releasing Hes1 through the promotion of the Hes1‐Hey2 complex	[33]
Nervous system	Inhibits oligodendrocyte differentiation	Inhibiting the transcriptional activity of Olig1/2 by binding to b‐HLH	[34]
Prostate	Regulates normal prostate development	Regulating androgen receptor binding to response elements on the NKX3.1 promoter	[35]
Gastric cancer	Tumor suppressor	Aberrant methylation of the ID4 promoter	[36]
Prostate cancer	Tumor suppressor	Modulating p53 transcriptional activity	[37]
B‐cell acute lymphoblastic leukemia	Oncogenic effects	Blocking the cell cycle and proliferation	[36]
Bladder cancer	Oncogenic effects	Blocking the cell cycle and proliferation	[38]

## 4 The Roles of ID4 in Cancers

The *Id4‐*KO mouse model is theoretically capable of successfully inducing a wide range of tumor models based on the indicative role of the results of a large number of retrospective studies; however, to date, only prostate carcinogenesis has been confirmed in the *Id4‐*KO mouse model [31]. The perspective of Id4's tumor suppressor activity is based on evidence that Id4 undergoes epigenetic gene silencing expression in mouse and human tumors including leukemia, rectal and gastric cancers [41, 42]. *P53* mutations are seen in about half of all tumors, and a large body of data suggests that about one‐third of prostate carcinogenesis is associated with *p53* mutations and aberrant transcriptional activity, whereas Id4 has been found to have a significant role in regulating *p53* transcriptional activity [37] (Table 1). In vitro assays with the WT‐prostate cancer cell line (LNCaP) and the *p53*‐mutant‐cell line (DU145) showed that Id4 significantly modulated *p53* transcriptional activity by inducing acetylation at the K373 position, thereby activating its downstream transcriptional activity [37].

**FIGURE 2:**
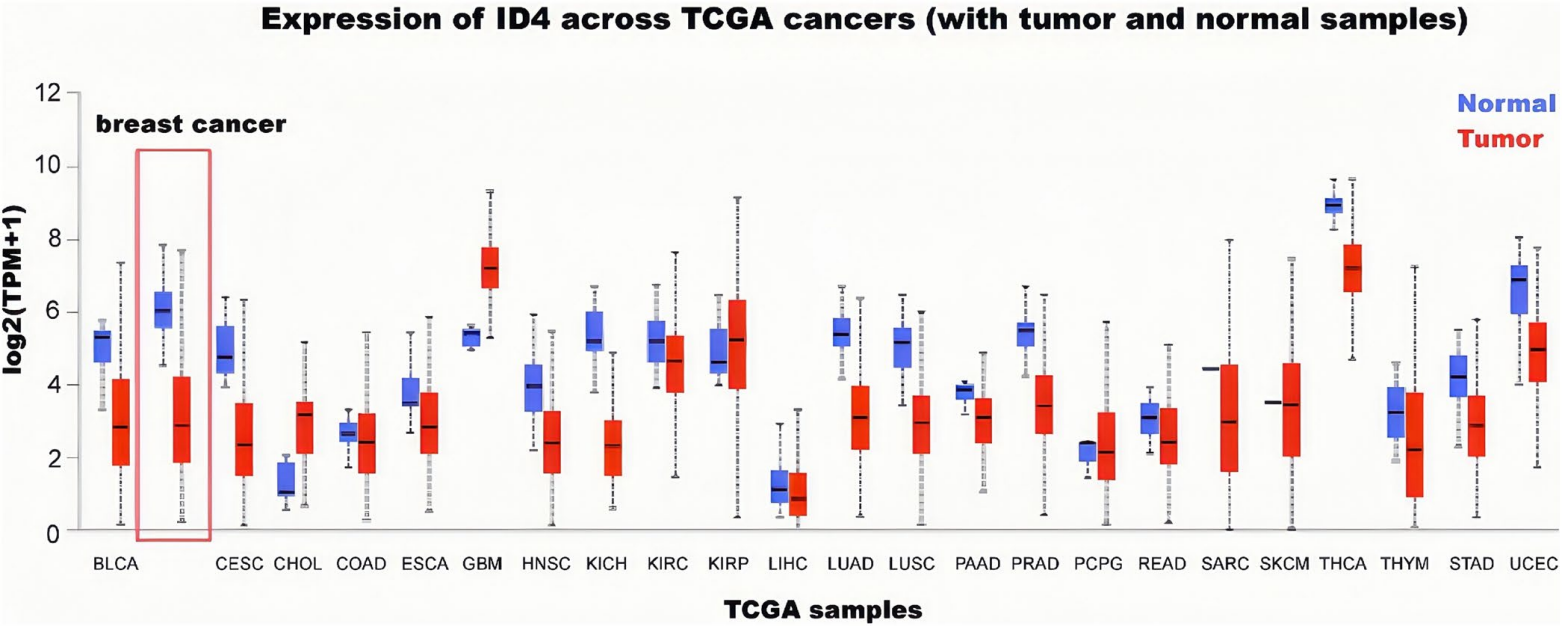
Expression of ID4 across TCGA cancers (with tumor and normal samples). The red rectangular box shows that the expression of ID4 was significantly lower in breast cancer than in normal tissues. BLCA, Bladder Urothelial Carcinoma; CESC, Cervical Squamous Cell Carcinoma and Endocervical Adenocarcinoma; CHOL, Cholangiocarcinoma; COAD, Colon Adenocarcinoma; ESCA, Esophageal Carcinoma; GBM, Glioblastoma Multiforme; HNSC, Head and Neck Squamous Cell Carcinoma; KICH, Kidney Chromophobe; KIRC, Kidney Renal Clear Cell Carcinoma; KIRP, Kidney Renal Papillary Cell Carcinoma; LIHC, Liver Hepatocellular Carcinoma; LUAD, Lung Adenocarcinoma; LUSC, Lung Squamous Cell Carcinoma; PAAD, Pancreatic Adenocarcinoma; PRAD, Prostate Adenocarcinoma; PCPG, Pheochromocytoma and Paraganglioma; READ, Rectum Adenocarcinoma; SARC, Sarcoma; SKCM, Skin Cutaneous Melanoma; THCA, Thyroid Carcinoma; HYM, Thymoma; STAD, Stomach Adenocarcinoma; UCEC, Uterine Corpus Endometrial Carcinoma.

However, some studies have noted that ID4 exhibited aberrantly activated pro‐oncogenic effects in bladder cancer and B‐cell acute lymphoblastic leukemia [36, 38, 43, 44]. Aberrant expression of ID4 blocks the cell cycle and proliferation, which correlates with increased expression of the cell cycle protein‐dependent kinase inhibitors p21 and p27 [36]. According to the above studies on the role of ID4 in various disease models, it can be seen that the process of cellular tissue differentiation is regulated by the expression level of ID4 and, remarkably, bone dysplasia and central nervous system developmental abnormalities are closely related to the abnormal expression of ID4. Meanwhile, studies on various tumor models have pointed to the tumor‐regulating role of ID4. Although some studies have not reached a consensus on whether *ID4* acts as an oncogene or suppressor gene, the relevance of some tumors, such as prostate cancer and leukemia, to the regulatory mechanism of ID4 has been supported by a large amount of data and interventions to modulate the expression of ID4 have been applied in clinical experiments. The mechanism of ID4 functions in other diseases needs to be further elucidated with the application of genomics, transcriptomics, and metabolomics. As shown in Figure 2, we analyzed the expression of ID4 in different cancers and normal tissues using the TCGA database. We found that the expression of ID4 was significantly lower in breast cancer than in normal tissues.

## 5 ID4 Regulates Mammary Gland Development

The role of the Id proteins has been studied to a limited extent in mammary gland development. Id1 is unnecessary for mammary gland development [45], whereas Id2 is necessary for normal RANK signaling within the mammary gland [46]. Id4 may play a more significant role in mammary gland development compared to other Id proteins. By observing the global *Id4‐*KO mouse model, researchers found that although the phenotype of abnormal mammary tissue development in mice can be observed, the effect of Id4 on mammary tissue in developing mice is difficult to observe dynamically in the knockout model due to its high mortality rate [31]. However, by histological observation of mammary structures in surviving 6‐week‐old mice, researchers found that ductal branching in the *Id4‐*null mice was significantly reduced until approximately 25 weeks old to fill the fat pad with an obvious reduction in branching, whereas there was no significant difference in mammary development between *Id4*‐null heterogeneous and WT mice, suggesting that a single copy of the *Id4* gene is sufficient to maintain mammary development in mice [47]. Researchers also found that Id4 was mainly expressed in the cap cells (specialized cells located at the tip of terminal end buds in the developing mammary gland) and basal cells of the mammary gland, and Id4 maintains mammary cell survival by inhibiting p38MAPK [47]. The mammary gland developmental abnormalities in *Id4*‐null mice were most notable for the abnormalities in the branching of the milk ducts as well as the number and structure of terminal end buds (TEBs): disorganization of the capsule cell layer, separation from the somatic cells, and abnormalities in the number and distribution of stroma, collagens, smooth muscle proteins, and keratins [47]. Although the mammary tissue development of *Id4‐*null mice was slowed down with reduced branching and incomplete degeneration of TEBs, the mammary gland had no impairment of functional differentiation, which manifested as normal lactation [47]. These early findings using *Id4*‐KO mice to study mammary gland development suggested that Id4 has a critical regulatory role in the development of mammary glands.

**FIGURE 3:**
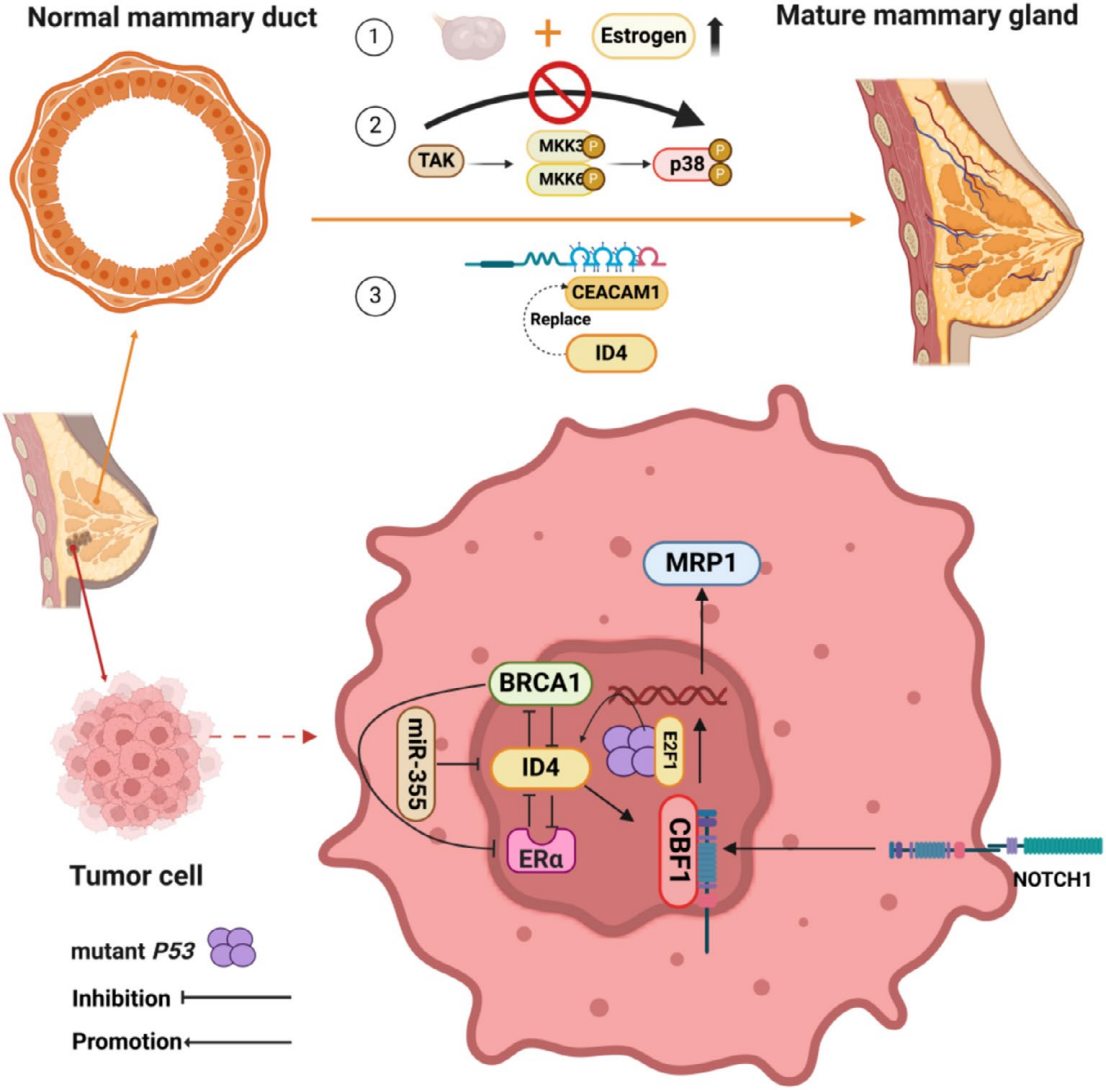
Diagram of the function of ID4 as a bridge linking mammary gland development and breast tumors. The top part of the figure represents that ID4 promotes mammary gland development through three major pathways. Among them, ①: ID4 can maintain ovarian function and promote estrogen expression; ②: ID4 can inhibit the p38MAPK pathway to maintain mammary cell survival; ③: ID4 can replace the CEACAM1 protein to repromote the formation of lumen in BC cells. The lower part of the figure illustrates the mechanism by which ID4 promotes breast carcinogenesis. These possible mechanisms include the interaction between ID4 and BRCA1, ERα, and participation in the NOTCH1 pathway. The protein complex of mutant p53‐E2F1 binds to a specific region of the ID4 promoter and promotes ID4 expression. Created in https://BioRender.com.

Several studies have further found that the *Id4*‐KO mice may compensatorily up‐regulate the expression levels of FOXA1 (Forkhead Box Protein A1) and estrogen receptor (ER)/progesterone receptor (PR), showing that the *Id4*‐KO mice's mammary gland showed significantly higher expression of ER/PR and FOXA1 in basal and luminal cells [48]. However, in the same model, the researchers found that the ovaries showed a reduction in granulosa cells and significant estrogen synthesis deficits, and both ovarian dysfunction and reduced estrogen could significantly affect the development of mammary tissue and the expression of ER/PR in mice [48]. This hypothesis was indirectly explained by Dong et al., who found that mammary cells from *Id4*‐null mice exhibited normal responses to estrogen and progesterone stimulation, including cell proliferation capacity [47]. According to the morphology of breast development, intraglandular lumen formation is a characteristic structural alteration of breast development, whereas degeneration or absence of lumen formation occurs in breast carcinogenesis, and researchers found that overexpression of ID4 can replace the CEACAM1 protein to repromote the formation of lumen in BC cells, and this evidence suggests that the role of ID4 in breast tissue has a correlative connection in mammary gland development and breast carcinogenesis [49] (shown in Figure 3).

Based on the above findings from a large number of mouse models of mammary development, it can be consistently concluded that Id4 expression is indispensable in mammary differentiation and development and primarily regulates the proliferation and differentiation of mammary basal cells and luminal epithelium, which are critical links in breast carcinogenesis.

## 6 **ID4** Regulates Breast Carcinogenesis

The mammalian mammary gland develops not only during the embryonic period, but also during postnatal with significant differentiation, development, and remodeling. Many studies have demonstrated that multiple cross‐cutting regulatory mechanisms regulate breast carcinogenesis and mammary development, such as the NOTCH and Wnt signaling pathways [17, 18]. The review indicated that the aberrant expression of several signaling pathways that regulate mammary development, including the Notch signaling pathway, can cause mammary developmental disorders and BC, including silencing and overactivation [17]. Of note, the regulation of the NOTCH pathway was found to interact with ID4 expression with the underlying mechanism to be uncovered [50]. One of the confirmed mechanisms [51] might be that ID4 contributes to breast carcinogenesis and chemotherapy resistance via the CBF1 (C‐promoter binding factor‐1, a critical downstream transcriptional factor in NOTCH1 signal pathway) and MRP1 (a chemo‐resistance related protein) signaling axis (shown in Figure 3). Early in 2009, researchers noted that while molecular phenotyping was useful in predicting the prognosis of BC subtypes and appropriate therapeutic strategies, detecting the origin of the tumor cells within the BC and targeting the developmental stage of these “cells of origin” was more instructive, based on the discovery of the biology of stem and progenitor cells within the mammary tissue [52]. Therefore, exploring key genes or proteins with critical regulatory roles under different models of both mammary gland development and breast carcinogenesis becomes a potentially effective means of intervening in BC.

*BRAC1* was the first oncogene identified in the breast and ovary, showing inherited mutations of *BRAC1* in the hereditary BC population and significantly reduced BRAC1 expression levels in sporadic BC patients [53], and as early as 2001, Beger et al. screened out the gene *ID4* as a key upstream regulator of *BRAC1* by using a ribozyme‐library‐based inverse genomics approach [54]. Subsequently, in 2003, using the rat carcinogen‐induced BC model, researchers found that BC‐inducing chemicals significantly increased the expression level of Id4 in mammary epithelial cells [55]. This evidence strongly suggested that breast carcinogenesis was accompanied by significant changes in ID4 expression level and, of particular importance, that the BC geographic oncogene *BRAC1* was strongly associated with ID4 expression. While *BRAC1* genetic mutations are not significantly associated with nonhereditary BC, data suggested reduced or undetectable levels of *BRCA1* mRNA in sporadic BC, ovarian cancer, and BC cell lines [54]. Analysis of clinical samples [56] demonstrated that *ID4* is amplified and overexpressed at a higher frequency in *BRCA1*‐mutant basal‐like breast cancer (BLBC) compared with sporadic BLBC. A study suggested that *BRCA1*‐loss BLBC originated from luminal epithelial progenitor cells rather than from basal stem cells. BC caused by specific knockdown of *Brac1* from mammary basal stem cells did not behave in the same way as human *BRAC1*‐associated BC or sporadic BLBC, whereas BC caused by specific knockdown of *Brac1* from luminal epithelial cells behaved in a typical BLBC [57]. In the context of BC, overexpression of Id4 is strongly associated with the TNBC [58, 59] and is negatively correlated with the expression of BRCA1 and ERα [60]. In 2012, researchers suggested that high expression of Id4 protein may be related to BRCA1 downregulation in TNBC, and TNBC with Id4 overexpression showed a basal‐like phenotype, consistent with the fact that BLBC is associated with low‐BRAC1 expression [59]. In a large number of subsequent studies, the reciprocal regulatory mechanisms of *ID4* and *BRAC1* have also been gradually revealed, such as Id4 negatively controlling the expression of Brca1 [54], and the *BRAC1*‐positive regulator miR‐335 downregulating the expression of ID4 (Figure 3) [61]. Furthermore, silencing *ID4* led to an increase in BRCA1 expression and resulted in a less aggressive phenotype in the MDA‐MB231 cell lines. The result of a study [56] from proteogenomic analysis of ID4 in BLBC indicated that ID4 localizes DNA at sites of active transcription and DNA damage, bridged through its biochemical interaction with the DNA damage response machinery, namely MDC1 (mediator of DNA damage checkpoint protein 1). Through MDC1, ID4 interacts with other DNA repair proteins (γH2AX and BRCA1) at fragile chromatin sites [56]. These results suggested a role for ID4 in the DNA damage response rather than regulation of transcription at these sites. However, the mechanism of interaction between ID4 and BRAC1 has not been fully elucidated, and even conflicting conclusions between some studies have been observed; thus, more in‐depth studies are urgently needed.

**FIGURE 4:**
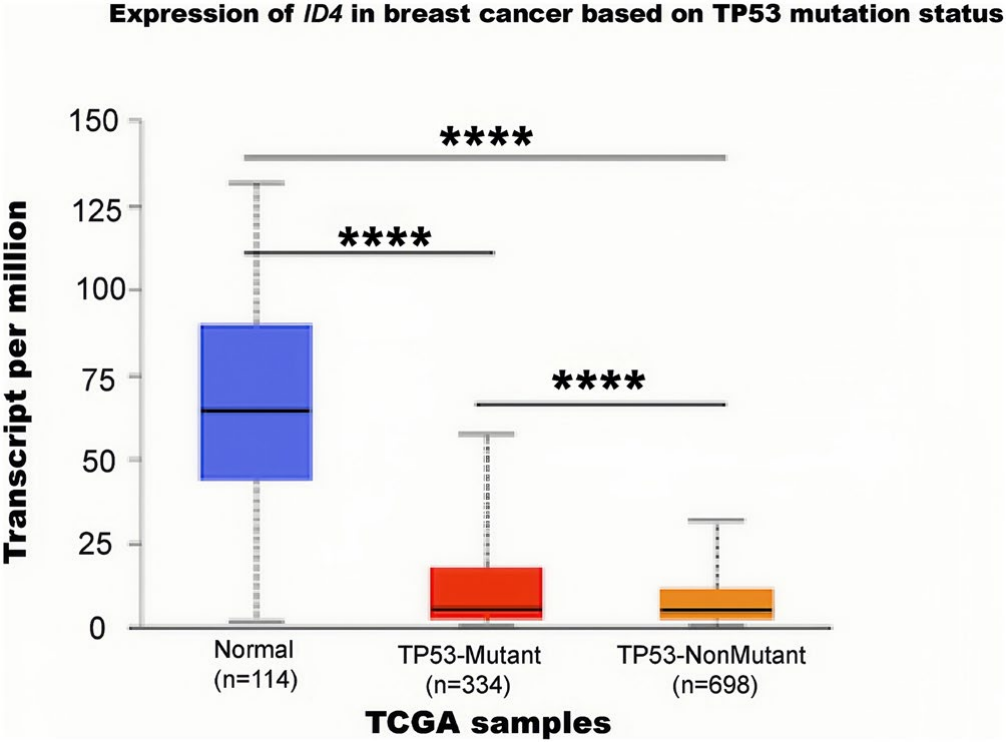
Expression of ID4 in breast cancer based on TP53 mutation status. Data were derived from breast cancer samples in The Cancer Genome Atlas (TCGA).

In BC cell lines SKBR3 and MDA‐MB‐231, ID4 is found to interact with the *p53* mutants R175H and R280K, facilitating angiogenesis [62]. Conversely, in MCF7 cells with wild‐type *p53*, this interaction is absent [62]. The underlying mechanism is that the protein complex of mutant p53‐E2F1 binds to a specific region of the *ID4* promoter and promotes *ID4* expression [62] (Figure 3). In highly aggressive TNBC, MALAT1 is associated with the degree of malignancy, and the researchers noted that in *p53*‐mutant TNBC, the cancer cells gain‐of‐function express VEGFA isoforms, and the ID4‐p53 complex has a role in regulating the recruitment of lncRNA MALAT1, which in turn regulates the degree of malignancy of TNBC [63]. Subsequent research [64] revealed that the lncRNA MALAT1 and the ID4 protein promote the back‐splicing of VEGFA exon 7, resulting in the formation of the circular RNA circ_0076611. This circular RNA interacts with various proliferation‐related transcripts, including MYC and VEGFA mRNAs, thereby enhancing the proliferation and migration of TNBC cells. However, a meta‐analysis showed that there was no significant correlation between *ID4* expression and *p53* mutation in BC, and this conclusion was validated in the TCGA data [65] (Figure 4).

**FIGURE 5:**
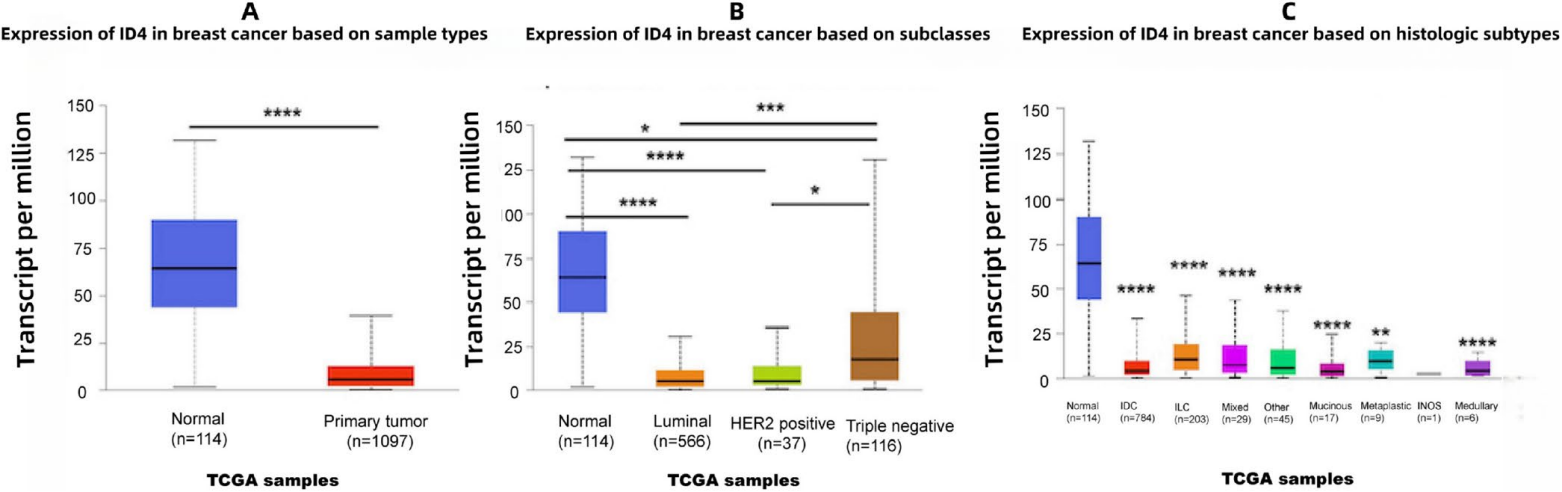
Analysis of ID4 expression in breast cancer. It illustrates the expression levels of the ID4 gene across distinct feature‐based classifications of breast cancer: (A) sample types (e.g., tumor tissue vs. normal tissue), (B) molecular subtypes (e.g., Luminal A, Luminal B, HER2+, Triple negative), and (C) histologic subtypes (e.g., ductal carcinoma, lobular carcinoma). The vertical axis represents transcript abundance in transcripts per million (TPM). Data were derived from breast cancer samples in The Cancer Genome Atlas (TCGA). IDC, invasive ductal carcinoma; ILC, invasive lobular carcinoma; INOS, invasive carcinoma not otherwise specified. *p < 0.05, **p < 0.01, *** or ****p < 0.0001.

In addition to the correlation between ID4 and the oncogene *BRAC1* and the tumor suppressor *p53*, which has been studied in depth, the correlation between ID4 expression status and the ER in BC has also been emphasized. Early in 2006, Paola de Candia et al. detected *ID4* mRNA in BC tissues of different pathological types and found that *ID4* mRNA levels were related to the distribution and density of ER, independently of Her‐2 status, and that *ID4* mRNA was present in nontumor breast tissues and ER‐negative mammary epithelium, whereas ER‐positive cells were *ID4* negative [58]. This evidence suggested that *ID4* may act as a tumor suppressor gene in ER‐positive BC and may be regulated by estrogen. ID4 is not expressed in ERα‐positive atypical ductal hyperplasia, ductal carcinoma in situ, and invasive carcinoma, but is present in ERα‐negative mammary epithelial cells, suggesting that ERα might negatively regulate ID4 (Figure 3) [58, 60]. ID4 expression is increased in BLBC/TNBC, but not in non‐TNBC [66, 67, 68]. However, in a meta‐analysis, there was no significant correlation between ID4 statues and BC subtypes [69]. TCGA database analysis showed that ID4 expression was different in different molecular and pathological subtypes (Figure 5). Further comprehensive studies are needed to reveal the correlation between ER status and ID4 expression.

The latest research [70] suggested that ID4 expression in BC cells is linked with the activation of motility pathways and enhances the production of VEGFA. This enhancement facilitates a paracrine interaction between VEGFR2 and integrin β3. Such interactions trigger the focal adhesion pathway downstream, promoting cell migration, invasion, and the formation of stress fibers [70]. This pathway has also been verified in other studies [71, 72], such as miR342 downregulating the expression of its target gene *Id4*, which further leads to the reduction of VEGF and Bcl2 (anti‐apoptotic)/Bax (pro‐apoptotic) ratio, thereby inhibiting the progression of TNBC.

**FIGURE 6:**
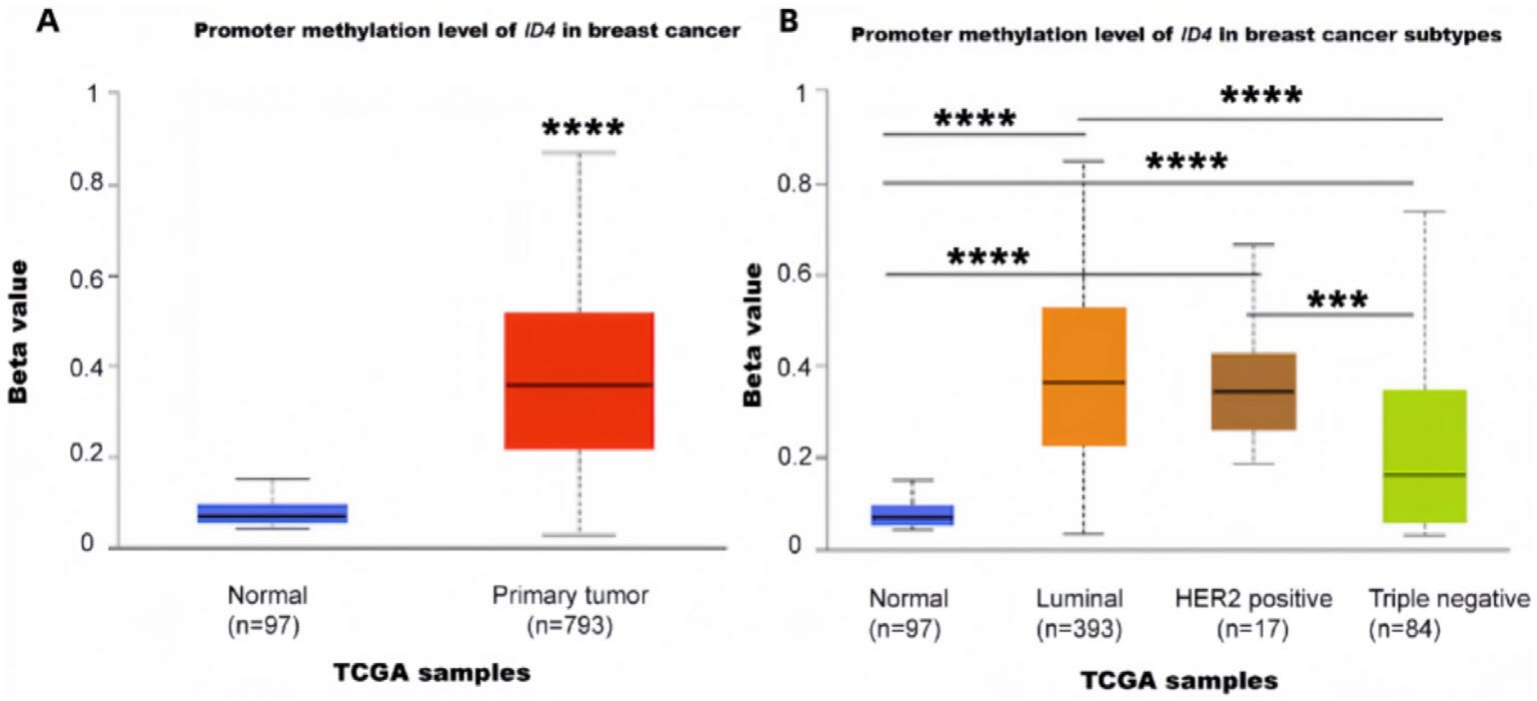
(A) Promoter methylation β values of ID4 in normal breast tissue (*n* = 97) versus primary breast tumors (*n* = 793). (B) Methylation β‐values of ID4 across breast cancer subtypes: Normal tissue (*n* = 97), luminal (*n* = 393), HER2‐positive (*n* = 17), and triple‐negative (*n* = 84). β‐values range from 0 (unmethylated) to 1 (fully methylated). Data derived from TCGA samples. *** or **** means *p* < 0.0001.

Meanwhile, researchers found that *ID4* is frequently silenced by promoter methylation in ER + BC and functions as a tumor suppressor gene in these tumors [73], and we also demonstrated that the luminal BC has a higher level of *ID4* promoter methylation using the TCGA database (Figure 6). Furthermore, researchers found that ER + BC with *ID4* hypomethylation were more likely to develop tamoxifen resistance [74], so interventions targeting the regulation of *ID4* methylation levels have potential function for treating endocrine‐resistant BC. In addition, *ID4* methylation status could serve as a prognostic biomarker in bc [75].

Using a melanoma model, the researchers found that the regulation of TGF‐β in the tumor immuno‐microenvironment is closely related to the expression of *ID4* and that the expression level of *ID4* significantly modulates the degree of immune infiltration, altering the tumor responsiveness to immunosuppressive therapy [76]. Donzelli et al. [77] showed a significant correlation between ID4 and macrophage marker CD68 protein expression in a range of triple‐negative breast tumors. Their further research [78] showed that activation of ID4 expression in tumor‐associated macrophages is observed as a consequence of BC cell paracrine activity and could participate in macrophage reprogramming in BC. Intervention in the immuno‐microenvironment of BC has been shown to have significant efficacy, so whether ID4 can similarly modulate the immuno‐microenvironment of BC needs to be further elucidated and is expected to be a means of combination immunotherapy.

Although we only discuss the role of ID4 in the occurrence and development of BC in this article, other ID proteins are also inevitably involved in this process, and understanding their roles may find new breakthroughs for ID4. For example, Id1 and Id3 have an important role in maintaining the cancer stem cell (CSC) phenotype in the TNBC subtype [79, 80, 81]. Similarly, ID4 is a key regulator of CSC self‐renewal and marks a subset of BLBC with a putative mammary basal cell of origin [82]. Not only that, Id1 may be a general negative regulator of anticancer drug‐induced apoptosis [83, 84], which revealed a joint role of Id4 and Id1 in BC development.

**FIGURE 7:**
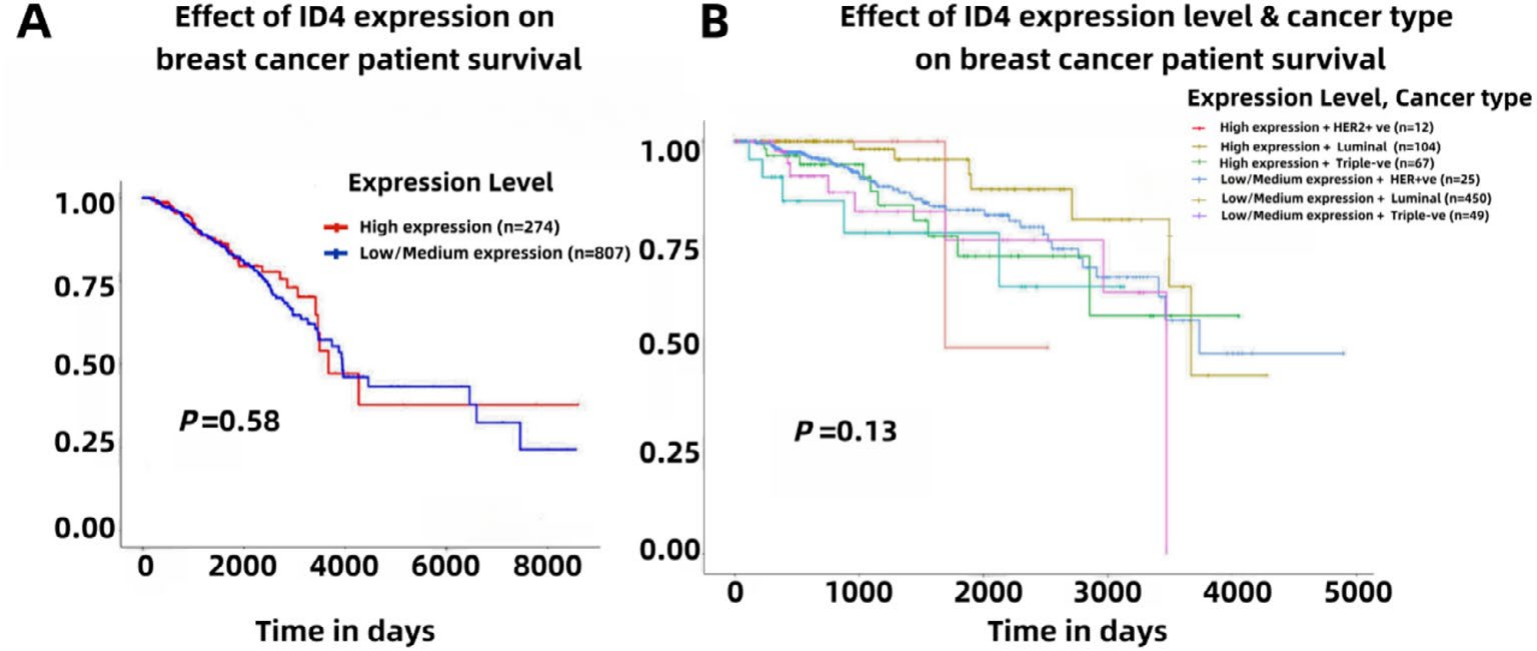
(A) Kaplan–Meier survival analysis comparing breast cancer patients with high ID4 expression (*n* = 274) versus low/medium ID4 expression (*n* = 807). (B) Survival analysis stratified by ID4 expression levels and cancer subtypes: High expression combined with HER2‐positive (*n* = 12), luminal (*n* = 109), or triple‐negative (*n* = 27), and low/medium expression combined with HER2‐positive (*n* = 35), luminal (*n* = 450), or triple‐negative (*n* = 49). Time is shown in days. Data derived from TGGA samples.

A study [85] showed that breast tumors with *ID4* overexpression presented significantly decreased rates of overall survival (OS, 69.4% vs. 82.2%; *p* = 0.013) and disease‐free survival (DFS, 61.2% vs. 86.3%; *p* < 0.001). *ID1* also presented markedly shorter OS and DFS rates. On the contrary, neither *ID2* nor *ID3* overexpression was associated with a worse prognosis in this subgroup of BC patients. Surprisingly, *ID4* did not significantly correlate with overall survival for these patients in the public database [85]. Similarly, we did not see a significant survival difference in the TCGA database (Figure 7).

## 7 Summary and Prospect

ID4 plays a pivotal role in various biological processes, particularly in cellular differentiation and cancer progression [1]. As a member of the transcriptional regulator family, ID4 lacks a DNA‐binding domain and functions primarily as a negative regulator of other b‐HLH proteins, leading to the formation of inactive heterodimers [4, 27]. This unique mechanism makes ID4 a critical player in embryonic development, where its expression decreases with cell differentiation [2, 3, 4]. ID4 is highly expressed in specific tissues, including the brain, thyroid, and mammary gland, and is implicated in osteoblast differentiation, neural precursor cell proliferation, and the regulation of several signaling pathways [20, 28, 30].

ID4 plays a crucial role in breast carcinogenesis by regulating the NOTCH1 signaling pathway and exhibiting reciprocal negative regulation with *BRCA1*, particularly in BLBC and TNBC [51, 54, 56]. Additionally, ID4 interacts with mutant *p53* to promote angiogenesis and is influenced by ER status, suggesting its potential function as a tumor suppressor in ER + bc [ 58]. Recent studies indicate that ID4 expression in BC activates motility pathways and enhances VEGFA production, promoting cell migration and invasion [70, 71, 72]. In ER + BC, ID4 is frequently silenced by methylation, functioning as a tumor suppressor and correlating with tamoxifen resistance, thereby highlighting its potential as a target for therapeutic intervention [74, 75]. Moreover, ID4 shows a strong correlation with macrophage marker CD68 expression, suggesting its role in macrophage reprogramming driven by paracrine signaling from BC cells [77, 78]. Notably, ID4's expression has prognostic implications, with overexpression associated with poorer survival outcomes in BC patients [85]. However, findings regarding its prognostic value remain inconsistent across studies and databases, necessitating further exploration.

The targeted inhibition of ID4 in breast cancer therapy remains an evolving field, with potential adverse effects largely dependent on the specific mechanisms of action of these agents. Given ID4's essential role in cellular differentiation and proliferation, its inhibition may disrupt tissue homeostasis, particularly in organs where it is highly expressed, such as the brain, thyroid, and reproductive system, potentially leading to neurodevelopmental abnormalities, thyroid dysfunction, or reproductive disturbances. As a key regulator of mammary gland development and estrogen receptor (ER) signaling, ID4 suppression may interfere with endocrine homeostasis, increasing the risk of menstrual irregularities, altered estrogen signaling, and other hormone‐related dysfunctions. Additionally, ID4 plays a critical role in shaping the tumor microenvironment, particularly in macrophage reprogramming and immune cell infiltration, suggesting that its inhibition could impair antitumor immunity or increase susceptibility to infections due to immune dysregulation. Moreover, ID4 exhibits a dual role in tumorigenesis, functioning as an oncogene in certain malignancies such as bladder cancer and leukemia while acting as a tumor suppressor in others, including ER‐positive breast cancer. Consequently, its targeted inhibition may lead to unintended oncogenic effects, particularly in patients with multiple cancer predispositions, highlighting the need for careful risk assessment. Furthermore, given its established role in chemotherapy resistance via the CBF1‐MRP1 pathway, ID4 inhibition may enhance chemosensitivity; however, it could also disrupt apoptotic pathways, resulting in unpredictable cellular responses. These potential risks underscore the necessity for a highly stratified approach in the clinical application of ID4‐targeted therapies, integrating molecular profiling to identify suitable patient populations and ensuring precise drug delivery to mitigate adverse effects.

Future research should focus on elucidating the multifaceted roles of ID4 in cancer biology and tissue differentiation, particularly in BC. Investigating the molecular mechanisms underlying ID4's regulatory functions could provide insights into its dual role as a tumor suppressor and an oncogene. Employing advanced genomic, epigenomics, transcriptomics, metabolomics, and lipidomics approaches will enhance our understanding of ID4's interactions with various signaling pathways and transcription factors, particularly in the context of tumor microenvironments [76, 77, 78].

Additionally, exploring the potential of ID4 as a therapeutic target in cancer treatment may yield significant clinical implications. The impact of ID4 on immune cell infiltration and its role in modulating the tumor immune microenvironment offer promising avenues for combination immunotherapy strategies. Understanding the mechanisms driving *ID4* methylation and expression in BC could lead to novel biomarkers for treatment resistance and recurrence [86, 87].

In summary, the continued investigation of ID4's biological functions and its role in BC will be essential for developing targeted therapies and improving prognostic accuracy in clinical settings.

## Author Contributions

Conceptualization: Y.S., P.Z., and S.B.; Writing – original draft preparation: Y.S., Y.Y., P.Z., and S.B. Writing – review and editing: Y.Y., Y.S., X.W., and H.Z. All authors have read and agreed to the published version of the manuscript.

## Consent

All authors read and approved the submission and final publication.

## Conflicts of Interest

The authors declare no conflicts of interest.

## Data Availability

The data sets used and analyzed in this study are available from the corresponding author upon reasonable request.
